# Expression and localization of CB1R, NAPE-PLD, and FAAH in the vervet monkey nucleus accumbens

**DOI:** 10.1038/s41598-018-26826-2

**Published:** 2018-06-06

**Authors:** Ryan Kucera, Joseph Bouskila, Laurent Elkrief, Anders Fink-Jensen, Roberta Palmour, Jean-François Bouchard, Maurice Ptito

**Affiliations:** 1 School of Optometry, University of Montreal, Montreal, QC Canada; 2 Faculty of Medicine, University of Montreal, Montreal, QC Canada; 3 Department of Psychiatry and Human Genetics, McGill University, Montreal, QC Canada; 4 St‐Kitts Behavioural Science Foundation, West Indies, Caribbean Saint Kitts and Nevis; 5 Laboratory of Neuropsychiatry, Psychiatric Centre Copenhagen, Copenhagen, Denmark

## Abstract

Extensive rodent literature suggests that the endocannabinoid (eCB) system present in the nucleus accumbens (NAc) modulates dopamine (DA) release in this area. However, expression patterns of the cannabinoid receptor type 1 (CB1R), the synthesizing enzyme *N*-acyl phosphatidylethanolamine phospholipase D (NAPE-PLD), and the degradation enzyme fatty acid amide hydrolase (FAAH) in the NAc have not yet been described in non-human primates. The goal of this study is therefore to characterize the expression and localization of the eCB system within the NAc of vervet monkeys (*Chlorocebus sabaeus*) using Western blots and immunohistochemistry. Results show that CB1R, NAPE-PLD, and FAAH are expressed across the NAc rostrocaudal axis, both in the core and shell. CB1R, NAPE-PLD, and FAAH are localized in medium spiny neurons (MSNs) and fast-spiking GABAergic interneurons (FSIs). Dopaminergic projections and astrocytes did not express CB1R, NAPE-PLD, or FAAH. These data show that the eCB system is present in the vervet monkey NAc and supports its role in the primate brain reward circuit.

## Introduction

The endocannabinoid (eCB) system is widely expressed in the central nervous system (CNS). It comprises the cannabinoid receptors type 1 (CB1R) and type 2 (CB2R), endogenous ligands (eCBs), and enzymes regulating the levels of eCBs^1–3^. The eCBs are lipophilic molecules that are synthesized “on demand” from the membrane of postsynaptic neurons after an increase in neural activity and calcium ion influx^1^. These endogenous ligands function as fast acting retrograde neuromodulators and are degraded rapidly^1^. The synthesis of anandamide, an eCB, is in part mediated by the release of N-acylethanolamines (NAEs) from N-acyl phosphatidylethanolamine (NAPE), by enzymes such as N-acyl phosphatidylethanolamine phospholipase D (NAPE-PLD)^4^. Its swift degradation is mostly mediated by the intracellular enzyme fatty acid amide hydrolase (FAAH)^5,6^. The expression of CB1R is found in many structures of the mouse, rat, monkey, and human brain, including the amygdala, cingulate cortex, prefrontal cortex (PFC), ventral pallidum, caudate, putamen, nucleus accumbens (NAc), ventral tegmental area (VTA) and lateral hypothalamus^7–10^. These regions are involved in reward, addiction and cognitive function. CB1R is also localized throughout the neocortex in rodents and primates^11–13^.

Neurophysiological studies first demonstrated that cannabis exerts its addictive potential from activating the pleasure-reward circuitry of the brain, namely the VTA that synapses with the NAc^14^. NAc dopamine (DA) elevation is qualitatively indistinguishable whether it is produced by THC, opioids, amphetamine, cocaine, ethanol, nicotine, barbiturates, or addictive dissociative anesthetics such as phencyclidine^15,16^. The prevalence of treatment for cannabis dependence is greater than treatment for cocaine addiction in the USA^17^, and its addiction potential has been further demonstrated by self-administration of THC in squirrel monkeys^17^. Within the NAc, there is a functional dissociation of the effect of VTA DA release onto the shell and core. The shell mediates feelings of reward while the core mediates locomotion toward rewards^18,19^ through a motor circuit that includes the substantia nigra (SN)^20^. While it is known that the eCB system may influence these circuits, the detailed anatomy of eCB system components in these circuits has not yet been fully described.

Recent investigations have intensified efforts on the localization of an endogenous cannabinoid system in the NAc. CB1R is localized in the NAc of rodents^11,21,22^, and is moderately expressed in the rat NAc^23^. It is also found in fast-spiking GABAergic interneurons (FSIs) in the NAc of mice^24^ and is expressed by GABAergic interneurons in the rat NAc^25^, but not in cholinergic or somatostatin-positive neurons in the rat NAc^25^ or dopaminergic neurons in the basal ganglia of rats^7^. In the rat NAc, CB1R is present on GABAergic medium spiny neurons (MSNs)^26,27^, and is also expressed in the mouse on the terminals of glutamatergic prefrontal cortical projecting neurons^28,29^. It has been proposed that cannabinoid receptors found on glutamatergic and GABAergic neurons modulate the activity of VTA DA neurons that project to the NAc^30^. Additionally, the SN has dopaminergic projections onto the NAc core^20^ which may be similarly modulated. In the rat, the SN receives GABAergic projections from CB1R containing neurons in the NAc^27^, suggesting an eCB role in the encoding of reward-related motor programs. The presence of CB1R has also been detected in the primate NAc^10^, but not thoroughly investigated.

NAPE-PLD and FAAH distributions in the monkey NAc remain both unknown since all detailed immunohistochemical studies available to date have been carried out in rodents. NAPE-PLD plays a role in the rodent NAc signaling^22^, and FAAH antagonists increase DA levels therein both with and without anandamide^31^. Furthermore, a large body of evidence shows that the eCB system modulates the neural activity within the NAc^14,30,32–34^. Since the NAc is a key player in addiction in rodent models and it contains components of the eCB system, it has been proposed that the latter may be involved in the mediation of addictive behavior. There is, however, no available data for the primate NAc and it is therefore the aim of this study to examine the expression and the precise localization of the eCB system components, namely CB1R, NAPE-PLD, and FAAH, in the vervet monkey NAc.

For its part, the CB2R is best known to be highly expressed in the immune system, including in brain microglia^35^, but more recently has been found at low levels in some neurons^3^. This includes the finding of CB2R genes and receptors to be expressed in mice midbrain DA neurons, and therein to effect DA neuronal firing and related behaviour^36^. However, its function in the CNS is not yet as well understood^3^ as CB1R on which the present investigation is focused. CB2R shares only 44% homology with CB1R^37^, and as a result functions significantly differently. CB2R shows little modulation of calcium channels or inwardly rectifying potassium channels in comparison to CB1R, which makes its signaling very different^38^. Its signaling is further complicated by species differences in CB2R response to identical drugs^3^. Despite common agonists, these receptors ultimately function differently. This is also reflected in differing affinity of their agonists. While 2-AG has high affinity at CB2R, anandamide serves as a weaker partial agonist of CB2R and has greater specificity to CB1R^3^. The significant difference in function of these two receptors makes them best studied separately. Here, the investigation of CB1R is complemented by the additional study of NAPE-PLD and FAAH, the synthesizing and degradative enzymes of anandamide.

## Results

### Western Blot Analysis

#### CB1R, NAPE-PLD, and FAAH presence and specificity in the NAc

**Figure 1:**
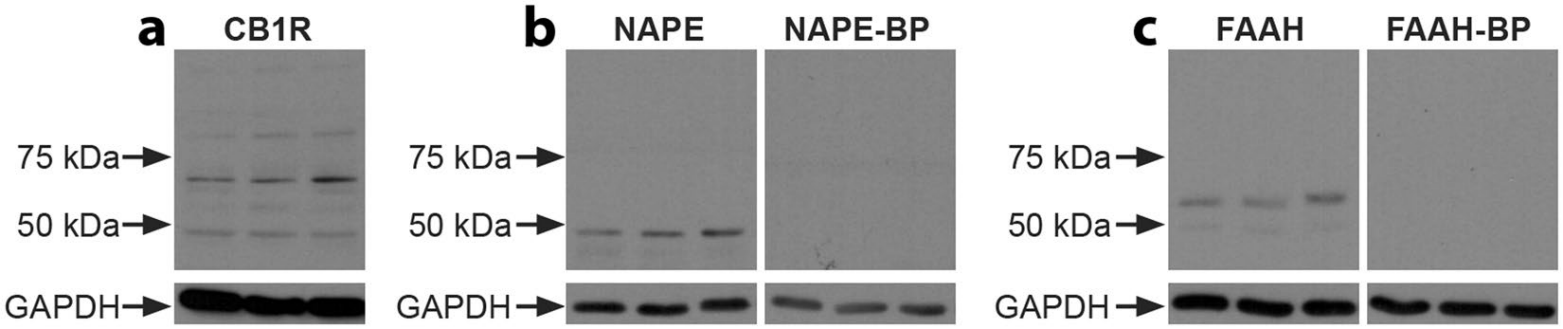
Presence of CB1R, NAPE-PLD, or FAAH in the NAc of three vervet monkeys. WB analysis of total protein samples for the CB1R antibody (**a**) showing detection of the expected major protein band at 60 kDa. For the NAPE-PLD antibody (**b**), the expected band is seen at 46 kDa, and not detected when pre-incubated with its blocking peptide. For the FAAH antibody (**c**), the expected band is seen at 63 kDa, and not detected when pre-incubated with its blocking peptide. All lanes contained 10 µg of total protein. The lower blots show the expression of GAPDH and demonstrate loading in all lanes.

We investigated the expression of three elements of the eCB system by evaluating the total amounts of CB1R and eCB-synthesizing (NAPE-PLD) and degradative (FAAH) enzymes in the monkey NAc. Immunoblots of three unfixed vervet NAc homogenates incubated with CB1R, NAPE-PLD, and FAAH antisera are shown in Fig. 1 and demonstrate their presence in the NAc. The specificity of the antibodies is shown by specific band recognition and blocking peptide signal abolishment. The CB1R blot recognized the expected major band at 60 kDa (Fig. 1a). The NAPE-PLD immunoblot shows as expected an intense band at 46 kDa (Fig. 1b), and the FAAH blot shows a dense expected band at approximately 63 kDa (Fig. 1c). Pre-incubation with the respective blocking peptides for NAPE-PLD and FAAH abolished the antibody signal for each (Fig. 1b,c), confirming the specificity of the antibody. However, for the CB1R antibody used here, a blocking peptide condition was not possible since there is not yet one commercially available. GAPDH loading controls for each immunoblot showed even levels of protein content across samples (n = 3) for each condition, as well as even loading between conditions with and without their respective blocking peptides. We provide here for the first time a set of results in primates that further extends the data obtained in rodents^23^.

### DAB Single Labeling

#### Delineation of core and shell in the NAc

**Figure 2:**
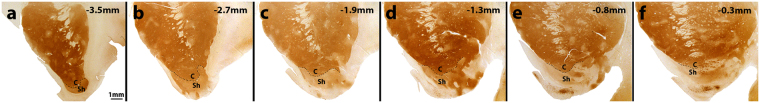
Photomicrographs of calbindin-stained coronal sections of basal forebrain across the rostrocaudal axis. (**a**–**f**) The first rostral section of the NAc was taken at approximately 3.5 mm anterior to the anterior commissure (AC) and the last caudal section was taken 0.3 mm anterior to the AC. The total length of the NAc was approximately 4 mm. Each slice distance relative to the AC is designated in the top right corner. The calbindin stain indicates the core and shell border of the NAc by overlaid dashed lines. The demarcation of core and shell is based on our own observation and previously published work^39,40^. Scale bar = 1 mm. C = core; Sh = shell.

To verify the precise location of the border between the core and shell of the NAc, DAB (3,3′-diaminobenzidine) immunostaining was carried out for calbindin-d28k (CB), a calcium binding and buffering protein that shows lower expression in the shell than the core^39^. Coronal sections of basal forebrain were taken, and 6 evenly spaced slices at a time were selected from across the rostrocaudal extent (Fig. 2). The border between the core and shell has been visualized with overlaid dashed lines. The demarcation of core and shell in the vervet monkey was found to be highly similar to previously published work in the macaque monkey^40^. Confirmation of the core and shell borders throughout the NAc allowed us to accurately determine the position of the nucleus and these subdivisions during confocal microscopy of our immunofluorescent experiments.

The vervet NAc has an irregular ovoid shape that varies across the rostrocaudal axis. The core expands as it progresses to the middle of the structure, and then becomes smaller once again as it reaches its caudal portion, and finally stretches into a thin oval as it subsides towards the most caudal extent. The shell encapsulates the ventral portion of the core throughout, always lying nearest to the apex of the heart-shaped striatum. The shell is largest in the middle of the rostrocaudal axis. The ventromedial beginning of the division of the core and shell can at times be clearly seen by the nearby ventricle reaching between them, particularly in mid-rostrocaudal sections.

#### Spatial expression of the CB1R system in the NAc

**Figure 3:**
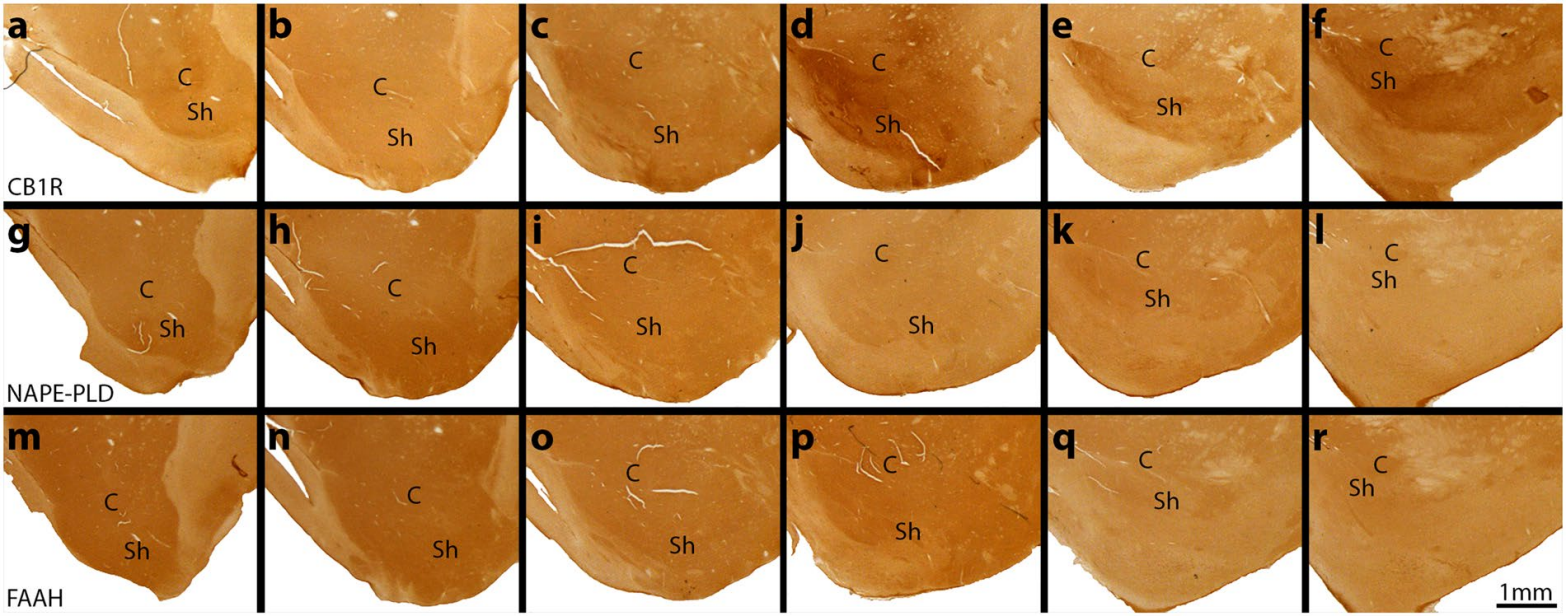
Spatial distribution of CB1R, NAPE-PLD, and FAAH throughout the rostrocaudal extent of the NAc. Coronal serial sections were taken adjacent to the CB stained slices in Fig. 2(a–f) Rostral sections show relatively homogenous staining of CB1R, but there is an increase of staining density in the medial portion of the shell beginning in mid-rostrocaudal sections (**c**). In (**d**) and further caudally, the CB1R expression is further increased in the medial shell. It is also noticeably augmented in the core and the ventral shell at these levels. (**g**–**l**) NAPE-PLD and (**m**–**r**) FAAH distributions remain relatively homogenous across the rostrocaudal extent. Scale bar = 1 mm. C = core; Sh = shell.

To visualize the localization of the CB1R system in the NAc, coronal serial brain sections containing the NAc were labeled with specific antibodies against CB, CB1R, NAPE-PLD, or FAAH. In the negative control condition, no primary antibody was used. Serial sections were taken from six representative levels across the rostrocaudal axis to compare the patterns of distribution. CB delimited anatomically the border between the core and the shell (Fig. 2a–f), as a reference for the rest of the series of slices, which were labeled for CB1R, NAPE-PLD, or FAAH. CB1R was detected throughout the NAc, but with higher expression in the dorsomedial and ventral shell in middle and caudal sections (Fig. 3c–f). In the caudal portion of the NAc, greater expression in the core was also present (Fig. 3d–f). NAPE-PLD and FAAH were homogeneously expressed across the rostrocaudal extent of the NAc (Fig. 3g–r). At low magnification, the entire NAc can be clearly seen and the distribution of eCB components visualized relative to the demarcation of the shell and core by CB. A consistent staining pattern across all monkeys was found.

### Immunofluorescent Double Labeling

#### CB1R, NAPE-PLD, and FAAH are expressed in medium spiny neurons (MSNs)

**Figure 4:**
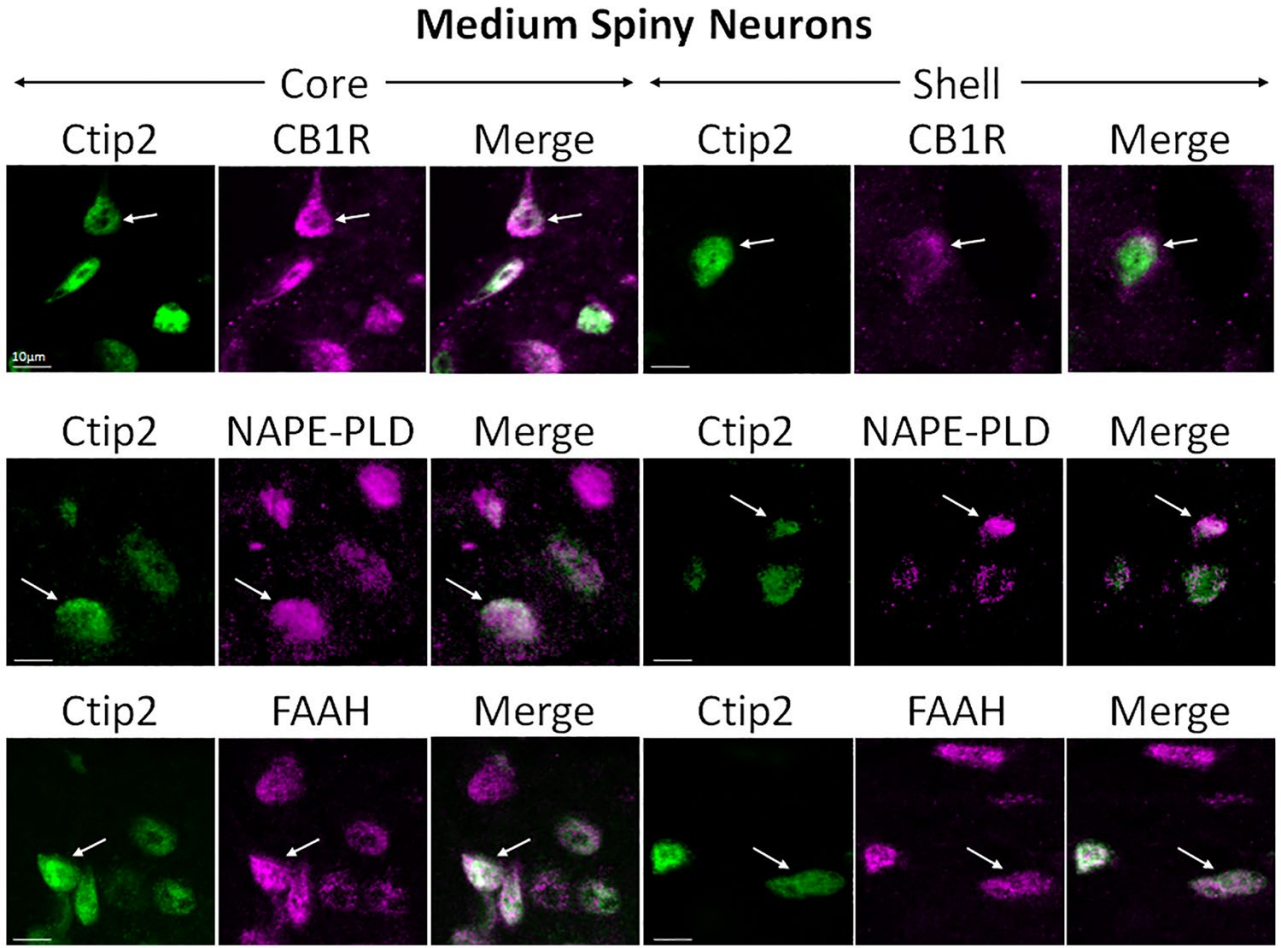
Double-label immunofluorescence illustrating co-localization of CB1R-IR, NAPE-PLD-IR, and FAAH-IR with Ctip2. Confocal micrographs of NAc co-immunolabeled for CB1R, NAPE-PLD, or FAAH (magenta), and Ctip2 (green), a specific marker for MSNs, in core and shell. Arrows point at Ctip2-positive MSNs that express either CB1R, NAPE-PLD, or FAAH. Scale bar = 10 µm.

MSNs were marked with Ctip2, a transcription factor specific for their differentiation^41^. Double immunolabeling was performed against CB1R, NAPE-PLD, and FAAH (Fig. 4). The three CB1R system components were all clearly expressed in the soma of Ctip2-positive neurons.

#### CB1R, NAPE-PLD, and FAAH are expressed in fast-spiking GABAergic interneurons (FSIs)

**Figure 5:**
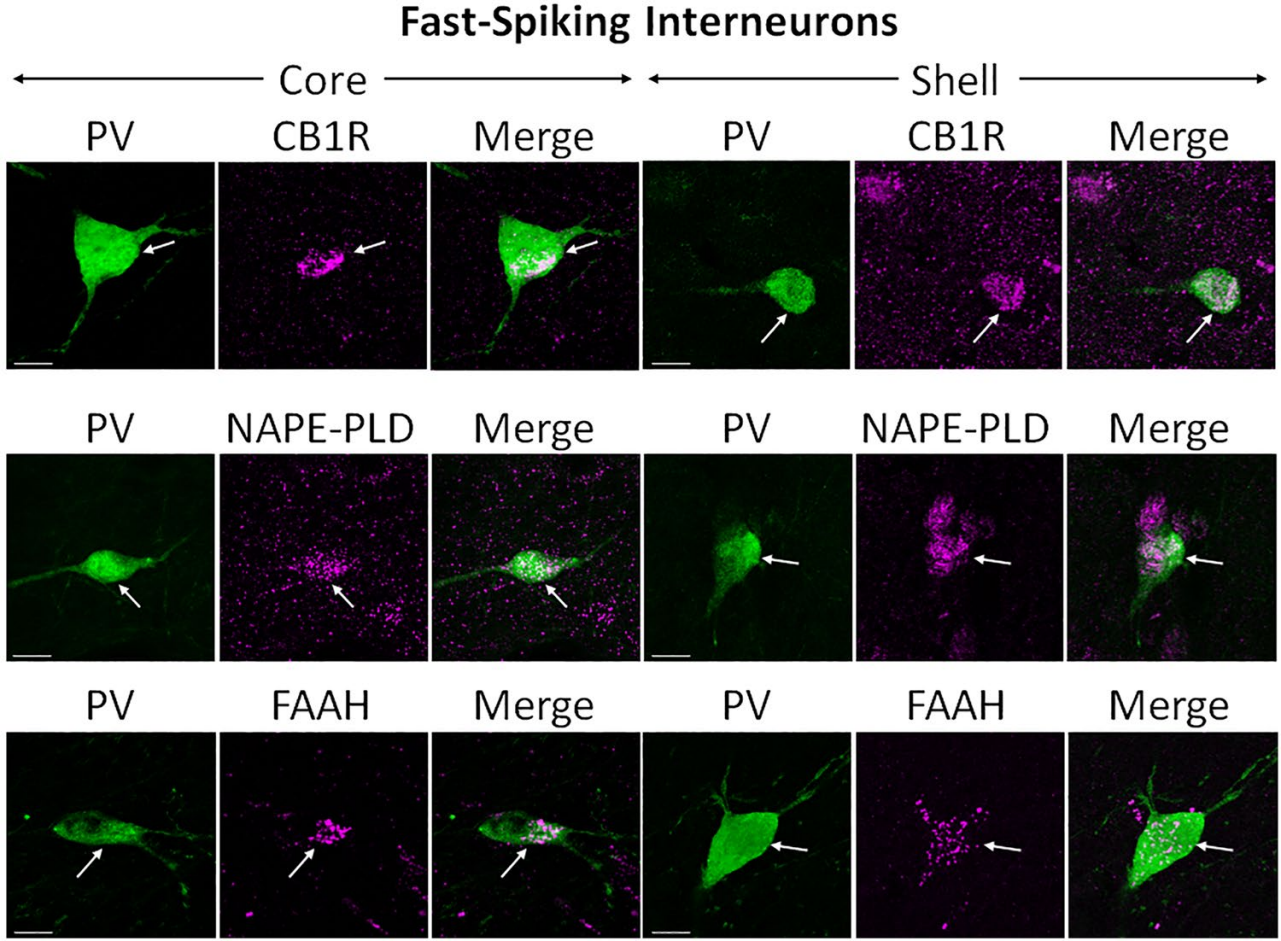
Double-label immunofluorescence illustrating co-localization of CB1R-IR, NAPE-PLD-IR, and FAAH-IR with parvalbumin (PV). Confocal micrographs of NAc co-immunolabeled for CB1R, NAPE-PLD, or FAAH (magenta), and PV (green), a specific marker for FSIs, in core and shell. Arrows point at PV-positive interneurons that express either CB1R, NAPE-PLD, or FAAH. Scale bar = 10 µm.

The calcium binding protein PV was used to mark FSIs^42,43^. Double immunolabeling was performed against the CB1R system components (Fig. 5). PV can be seen throughout perikarya and fibers, extending down to axons. The eCB components can be seen most clearly in the cell bodies.

#### CB1R, NAPE-PLD, and FAAH are not expressed in DA-producing cells

**Figure 6:**
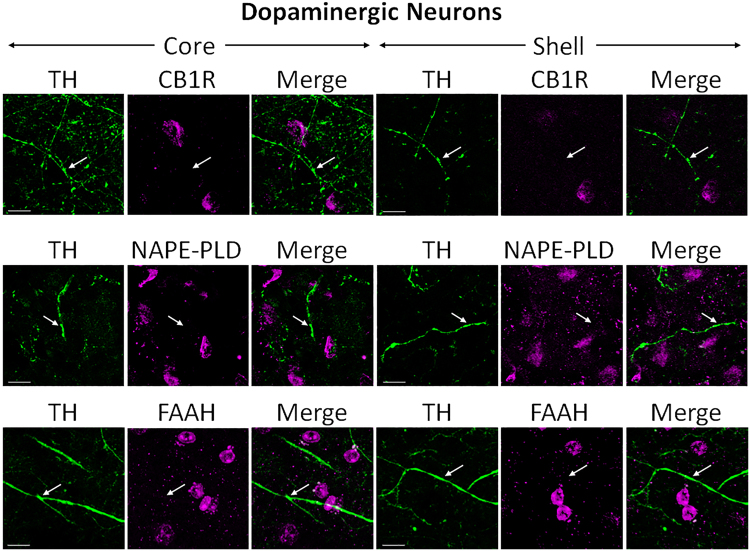
Double-label immunofluorescence illustrating co-localization of CB1R-IR, NAPE-PLD-IR, and FAAH-IR with tyrosine hydroxylase (TH). Confocal micrographs of NAc co-immunolabeled for CB1R, NAPE-PLD, or FAAH (magenta), and TH (green), a specific marker for dopaminergic projections, in core and shell. Arrows point at TH-positive axons and terminals that do not express either CB1R, NAPE-PLD, or FAAH. Scale bar = 10 µm.

Tyrosine hydroxylase, the rate-limiting enzyme in DA synthesis, was used as a specific marker of dopaminergic neurons. No co-localization was obtained when sections were stained with TH and CB1R (Fig. 6). Axon fibers and terminals stained with TH surround the multiple cell bodies labeled with CB1R, suggesting a complementary but not overlapping staining pattern. Sections immuno-stained with TH and NAPE-PLD or FAAH showed similar patterns of complementation without co-localization to that of TH with CB1R (Fig. 6). Lack of CB1R system expression in dopaminergic neurons is consistent with previous findings in rodents^7^.

#### CB1R, NAPE-PLD, and FAAH are not expressed in glial cells

**Figure 7:**
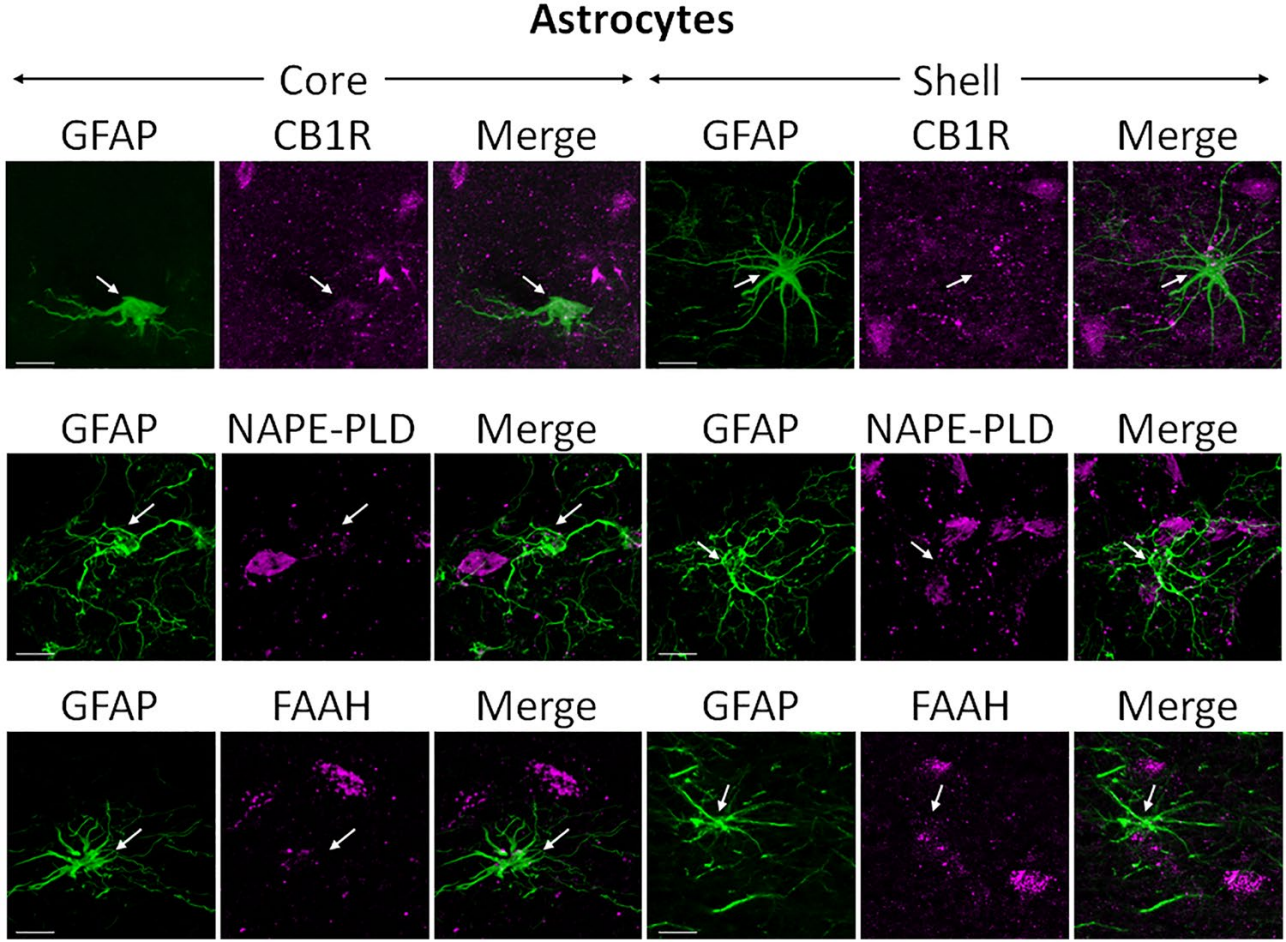
Double-label immunofluorescence illustrating co-localization of CB1R-IR, NAPE-PLD-IR, and FAAH-IR with glial fibrillary acidic protein (GFAP). Confocal micrographs of NAc co-immunolabeled for CB1R, NAPE-PLD, or FAAH (magenta), and GFAP (green), a specific marker for astrocytes, in core and shell. Arrows point at GFAP-positive glial cells that do not express either CB1R, NAPE-PLD, or FAAH. Scale bar = 10 µm.

Glial fibrillary acidic protein (GFAP) was used to mark astrocytes. CB1R, NAPE-PLD, and FAAH were not expressed in GFAP-positive glial cells in the NAc (Fig. 7). GFAP immunoreactivity was clearly detected; individual glial cell bodies and processes can be seen. While CB1R, NAPE-PLD, and FAAH were expressed in neurons, they did not co-localize with GFAP-positive cells (Fig. 7).

## Discussion

This study reports for the first time the expression and localization of CB1R, NAPE-PLD, and FAAH in the NAc of vervet monkeys. Immunoblots of vervet monkey NAc tissue against CB1R, NAPE-PLD, and FAAH antisera were similar to those previously reported for rodents^44,45^ and vervet monkey retinal and thalamic tissues^46,47^. The NAc can be anatomically separated into two distinct parts: the outer shell and interior core^48,49^. Each part plays a different role in behavior and addiction^50^. The core is responsible for major output onto the SN, and receives all inputs from the SN, as well as some inputs from the VTA onto its medial portion^20^. The shell largely projects to the VTA^51^, though the SN also receives minor projections from the lateral shell. The shell also receives many projections back from the VTA^52^, mostly onto its medial and ventral portions^20^. We found differences in CB1R expression in the core and shell at diverse points along the rostrocaudal axis, with a higher dorsomedial and ventral expression in the shell in middle and caudal sections, and increased core expression in mid-rostrocaudal sections of the NAc (Fig. 3). This may indicate a more pronounced role of eCBs in a circuit where the SN receives projections from the middle and caudal portion of the shell and the middle portion of the core. As for the VTA, the influence of eCBs stems from projections onto the middle portion of the NAc core, and its greater connections with the middle and caudal portions of the shell.

Recent research has shown that the NAc plays a key role in action selection; as such, abnormalities in accumbal signaling have been linked to the development of addictions and other neuropsychiatric conditions^53,54^. It has been hypothesized that DA transmission in the NAc is implicated in translating motivation into action^55^, and reinforcement learning^56^. Mesolimbic DA neurons projecting onto the NAc have two modes of firing, either “tonic” or “phasic”^57,58^, both of which are implicated in the development of drug addiction^59^. It has been reported that the eCB system plays a role in the modulation of both phasic and tonic DA firing in the NAc^60^. Although there seems to be moderate to low levels of CB1R in the NAc^9,11,61,62^, a collection of work has shown that the CB1R antagonists and agonists modulate DA NAc signaling, in both rodents and primates^63,64^. Additionally, CB1Rs in the monkey brain have been imaged *in vivo* using various radioligands^65,66^. CB1R is known to be responsible for the psychoactive effects of marijuana, the effects of which have been blocked by a CB1R antagonist in marijuana smoking humans^67^ and THC and anandamide self-administering monkeys^17^. This suggests the importance of CB1R in reward and addiction. CB2R might also play a role in the reward circuit. CB2R knockout mice have been shown to lack conditioned place preference for nicotine and to self-administer less nicotine^68^. A CB2R antagonist also blocked conditioned place preference from nicotine and reduced nicotine self-administration^68^. Interestingly, the CB2R agonist also reduced cocaine self-administration^69^. CB2R is also expressed in mouse VTA DA neurons that have reduced excitability in the presence of CB2R agonists and reduced cocaine self-administration^36^. However, the role of the CB2R in the reward circuit and in neurons has not been studied in as much detail as the CB1R, and the CB2R remains better known for its critical role in immune function in the brain^35^. For these reasons, we have focused our attention on describing the anatomy of CB1R expression in the NAc.

We have found that CB1R, NAPE-PLD, and FAAH are expressed in both cell bodies and processes in MSNs and FSIs, but not in dopaminergic projections or astrocytes. While it is well known that eCBs act as retrograde neuromodulators^70^, it has also been suggested that certain substrates, particularly anandamide, can act on CB1R postsynaptically or intrinsically^71^, or in an autocrine fashion^72,73^. Our results show the presence of CB1R in cell bodies, including on the cell membrane, which suggests that eCBs may also act as postsynaptic or autocrine modulators in the monkey NAc. This is further supported by the presence of CB1Rs in neuronal cell bodies and dendrites in the rat striatum^27^. Since the dopaminergic neurons which innervate the MSNs do not express the eCB system, any anterograde eCB modulation would likely come from FSIs or glutamatergic terminals from the PFC, though eCB spillover from nearby MSN or FSI dendrites is also possible^74,75^.

FSIs may act to synchronize the spike timing of larger populations of neurons^76^, such as MSNs. It has also been reported that FSIs may inhibit themselves^77^. The presence of the eCB system in FSIs suggests that it plays a role in how the spike timing of MSNs is regulated by FSIs, the decreased synchrony of which could lead to weaker inhibition of dopaminergic neurons in the VTA that project onto the NAc. Specific outputs of the NAc come from ensembles of neurons that are clustered spatially close to one another and fire in a coherent and synchronous manner, and require a strong excitatory input^78^, further supporting the importance of FSI synchronization of MSNs. The eCB system may also play a role in the gating of MSNs between their two possible resting potentials of a physiologically silent hyperpolarized “down” state and their slightly depolarized “up” state at which action potentials can be induced^79^. CB1R activation, whether on MSN cell bodies in the NAc or on MSN terminals in the VTA^30^ and SN^27^, may directly reduce inhibition of DA neuron firing in the NAc. Additionally, the presence of CB1Rs on NAc FSIs^24^, which are important for the synchronization of ensembles of MSNs, may further contribute to the regulation of MSN output^80^. CB1Rs have been detected on glutamatergic neurons terminating in the NAc in mice which suggest that they may also reduce MSN output^29^. Since some MSNs are also glutamatergic in addition to being GABAergic^81^, it may also be possible that CB1R affects glutamatergic signaling onto interneurons at the terminations of these MSN projections. Taken together, inhibition by CB1R activation on both GABAergic and glutamatergic cells may reduce the release of GABA by MSNs projecting onto VTA and SN DA neurons, which in turn may increase DA in the NAc and other brain regions. This dysregulation of DA release could enhance reward perception and motor pattern activation, underlying addiction. These results suggest that the eCB system may play a crucial role in the modulation of the primate brain reward circuit that remains to be investigated.

## Materials and Methods

### Animals

Ten vervet monkeys (*Chlorocebus sabaeus*) were used in this study (3 females and 7 males aged 0.4 years (y), 0.5 y, 0.75 y, 2 y, 2 y, 2 y, 2.5 y, 3 y, 5.5 y, and 11 y). The animals were born and raised in an enriched environment in the laboratories of the Behavioral Sciences Foundation (BSF; St-Kitts, West Indies), a facility that is recognized by the Canadian Council on Animal Care (CCAC). The tissue samples used in this study were donated by the BSF and were part of a wider project approved by the ethics committee at the Université de Montréal (Comité de Déontologie de l′Expérimentation sur les Animaux, Ref number: 17-097). They were utilized in accordance with the CCAC requirement for reduction of animals sacrificed for experimental purposes.

### Tissue Preparation

Brain sections that included the whole NAc were prepared following previously published methods^46,47,82^. Briefly, the animals were sedated with ketamine hydrochloride (10 mg/kg, i.m.), then euthanized with an overdose of sodium pentobarbital (25 mg/kg, i.v.) and perfused transcardially with 0.1 M phosphate buffered saline (PBS, 0.1 M) until complete exsanguination. The brain was then either rapidly frozen unfixed for Western blots (WB), or was bathed in a 4% paraformaldehyde solution in PBS for immunohistochemistry. The fixed brain was then stereotaxically blocked, removed from the skull, weighed, and the volume determined. The brain was finally cryoprotected in graded sucrose solutions and embedded in Shandon embedding media at −65 °C. The blocks were sliced (40 µm) with a cryostat in a serial manner and stored, again according to previously published methods^82^.

### Western Blotting

To test the presence and specificity of the CB1R, NAPE-PLD, and FAAH antisera, WB were performed on unfixed vervet NAc tissue from 3 different monkeys. The entire NAc from one hemisphere was dissected from each monkey and homogenized by hand using a sterile pestle in RIPA buffer (150 mM NaCl, 20 mM Tris, pH 8.0, 1% NP-40 [USB Corp., Cleveland, OH, USA], 0.5% sodium deoxycholate, 0.1% SDS, 1 mM EDTA), supplemented with a protease-inhibitor mixture (aprotinin 1:1,000, leupeptin 1:1,000, pepstatin 1:1,000, and phenylmethylsulfonyl fluoride 0.2 mg/ml); Roche Applied Science, Laval, QC, Canada). After the samples were centrifuged (4 °C, 10 minutes), the supernatant was extracted and content was equalized using Thermo Scientific Pierce BCA Protein Assay Kit (Fisher Scientific, Ottawa, ON, Canada). Ten µg of protein per well was loaded in a 10% sodium dodecyl sulphate (SDS)-polyacrylamide gel and electrophoresed. It was then transferred onto a nitrocellulose membrane filter (BioTrace NTll; Life Sciences, Pall, Pensacola, FL, USA) and washed 3 times 10 minutes in TBST (0.15 M NaCl, 25 mM Tris-HCl, 25 mM Tris, 0.5% Tween-20). It was blocked for an hour in 5% skim milk (Selection, Montreal, QC, Canada) in TBST, and left to incubate overnight in an IgG primary antibody raised in rabbit; anti-CB1R, anti-NAPE-PLD, and anti-FAAH at a concentration of 1:1,000 in blocking solution. For blocking peptide (BP) control conditions a ratio of 5:1 BP to antibody was pre-incubated for 1 hour before being diluted in blocking solution (final concentrations of NAPE-PLD 1:1,000, NAPE-PLD BP 1:200; FAAH 1:1,000, FAAH BP 1:200). On the following day, 6 washes in TBST of 5 minutes each preceded and followed incubation of the blot in secondary antibody conjugated to horseradish peroxidase (1:5,000; Jackson Immunoresearch, West Grove, PA, USA) in blocking solution for two hours. The blot was washed 6 times 5 minutes in TBST. Detection was done using a homemade ECL WB detection reagent (final concentrations of 2.50 mM luminol, 0.4 mM p-coumaric acid, 0.1 M TrisHCl, pH 8.5, 0.018% H_2_O_2_). After detection, the loading control was performed. The blot was washed 3 times 10 minutes in TBST, blocked for an hour in 5% skim milk in TBST, then incubated overnight in an anti-GAPDH IgM primary antibody raised in mouse at a concentration of 1:20,000. The next day, the blot underwent the same washes, incubation in secondary antibody, washes again, and detection, as above.

### DAB immunohistochemistry

DAB immunostaining was performed in free-floating solution similarly to previously published methods^47^. Briefly, brain sections of 40 μm that included the NAc were cleaned 3 times for 10 minutes each in washing solution (0.1 M PBS buffer pH 7.4, 0.03% Triton X-100). The tissue was then protected from non-specific binding in a blocking solution (0.5% triton, 10% either normal donkey serum or normal goat serum, in 0.1 M PBS) for 90 minutes. The tissue was then placed in primary antibody (Table 1) diluted in blocking solution and left to incubate overnight at 4 °C. After washing the sections for 10 minutes once and 5 minutes twice in washing solution, the slides were incubated in secondary antibody (biotinylated goat anti-rabbit, donkey anti-rabbit, or donkey anti-mouse diluted 1:200 in blocking solution) for 2 hours. Tissue was then washed 3 times for 10 minutes and incubated for 1 h in an avidin-biotin-conjugated horseradish peroxidase (Vectastain ABC kit, Burlingame, CA, USA) solution (1:500 in 0.1 M PBS). Another 3 washes of 10 minutes were performed and the sections were treated with a DAB substrate, until the tissue was coloured (1 to 10 minutes). The tissue was then washed again for 3 times of 10 minutes and the sections were mounted on gelatinized slides and left to dry. They then underwent dehydration in graded ethanol, were cleared in xylene, and cover slipped with Permount mounting media (Fisher Scientific; Pittsburgh, PA, USA).

**Table 1:** Primary antibodies used in this study.

Antibody	Immunogen	Source	Working Dilution	RRID
CB	Recombinant protein specific to amino terminus of human CB	Cell Signaling Technology, Danvers, MA, USA	DAB 1:500, IF 1:200	AB_2687400
CB1R	Fusion protein containing aa 1–77 of rat CB1R	Calbiochem, Gibbstown, NJ, USA	DAB 1:300, IF 1:200, WB 1:1,000	AB_211563
CTIP2	Synthetic peptide corresponding to aa 1–150 of human Ctip2	Abcam plc., Cambridge, UK	IF 1:200	AB_2064130
FAAH	Synthetic peptide corresponding to aa 561–579 of rat FAAH	Cayman Chemical, Ann Arbor, MI, USA	DAB 1:200, IF 1:200, WB 1:1,000	AB_10078701
GFAP	GFAP purified from pig spinal cord	Cell Signaling Technology, Danvers, MA, USA	IF 1:200	AB_561049
NAPE-PLD	Synthetic peptide from human NAPE-PLD aa 159–172	Cayman Chemical, Ann Arbor, MI, USA	DAB 1:200, IF 1:200, WB 1:1,000	AB_10507996
PV	Parvalbumin purified from carp muscle	Swant, Marly, Fribourg, Switzerland	IF 1:200	AB_10000343
TH	TH purified from PC12 cells derived from rat pheochromocytoma; recognizes an epitope on the outside of the regulatory N-terminus of TH	EMD Millipore, Chemicon, Temecula, CA, USA	IF 1:200	AB_2201528

CB: Calbindin-d28k; CB1R: cannabinoid receptor type 1; CTIP2: CTIP2 transcription factor; DAB: 3,3′-diaminobenzidine immunostaining; FAAH: fatty acid amide hydrolase; GFAP: glial fibrillary acidic protein; IF: immunofluorescence; NAPE-PLD: N-acyl phosphatidylethanolamine-specific phospholipase D; PV: parvalbumin; TH: tyrosine hydroxylase; WB: Western blot.

### Immunofluorescence

Double-labeling were performed on the vervet monkey NAc, following previously published methods in the retina and dorsal lateral geniculate nucleus^47,83^, but with minor changes. Tissue was treated the same as in the above DAB protocol for “day one”, until primary antibody incubation. When the tissue was ready to be incubated in primary antibody, it was exposed to two primary antibodies at dilution rates mentioned in Table 1 and incubated overnight. On the second day, the tissue was washed in washing solution for 3 times 10 minutes. The tissue was then incubated in secondary antibody diluted in blocking solution (1:200). The slices were washed 3 times for 10 minutes in 0.1 M PBS, then 1 time for 10 minutes in 0.1 M PB. They were then mounted onto gelatinized slides and left to dry for approximately half an hour before coverslipping using Fluoromount G mounting medium (SouthernBiotech, Birmingham, AL, USA).

## Equipment and Settings

### Brightfield Microscopy

DAB slides were analyzed under a Leica microscope, using a 0.65X objective. The images were taken in Qcapture (Micro-Bright Field) software. All adjustments, such as size, colour, brightness and contrast, were performed using ImageJ and Adobe Photoshop (CS6; Adobe Systems; San Jose; CA, USA) and subsequently exported onto Adobe InDesign (CS6; Adobe Systems), where the final figure layout was completed.

### Confocal Microscopy

Fluorescence was detected using a Leica TCS SP2 confocal laser scanning microscope with default Leica software (Leica Microsystems, Exton, PA, USA). Images were taken under a 63X objective, at resolutions of either 1080 × 1080 or 2160 × 2160 pixels. Green and far-red channels were used to detect images from the 40 µm slices. The green channel (488 nm) was used to detect cell markers and the far-red channels (647 nm) to detect CB1R, NAPE-PLD, and FAAH. To enhance some images, z-stacks were taken for optimization and averaged using ImageJ. Z-stacks allowed for visualization of cells along the X-Y, X-Z and Y-Z axes. All adjustments, such as size, colour, brightness and contrast, were performed using ImageJ and Adobe Photoshop CS6 and subsequently exported onto Adobe InDesign CS6, where the final figure layout was completed.

## Antibody Characterization (for more info, please see Table 1)

### CB

A monoclonal mouse anti-calbindin-d28k (CB, Cell Signaling Technology, Danvers, MA, USA, Cat# 13176, RRID: AB_2687400) was developed with a recombinant protein specific to the amino terminus of human CB. CB labels cell bodies, dendrites and their spines, and axons and their terminals, of MSNs in the basal ganglia of the monkey and rat, with the most intense labeling occurring in the matrix of the cytoplasm^84^. Primary antibody working dilutions and other detailed information are included in Table 1.

### CB1R

A polyclonal rabbit anti-CB1R (CB1R, Calbiochem, Gibbstown, NJ, USA, Cat# 209550-100UL, RRID: AB_211563) was developed using the first 77 amino acid residues of rat CB1R. A major 60 kDa band in rat heart tissue^85^, and minor 23, 72 and 180 kDa bands from various other tissues (manufacturer data sheet) are recognized by this antibody. It has been previously reported that this antibody is specific, using a CB1R knockout mouse retina^45^. It recognizes CB1R in other species, including the vervet monkey^46^.

### CTIP2

A monoclonal rat anti-Ctip2 antibody (Ctip2, ab18465, Abcam plc., Cambridge, UK, Cat# ab18465, RRID: AB_2064130) was developed using a synthetic peptide corresponding to amino acid 1–150 of the human Ctip2. It is a specific marker of GABAergic medium-sized spiny neuron (MSN) differentiation, which comprises over 90% of striatal neurons, and is not present in interneurons^41^. This antibody’s use has been verified in primates^86^.

### FAAH

A polyclonal rabbit anti-fatty acid amide hydrolase (FAAH, Cayman Chemical, Ann Arbor, MI, USA, Cat# 101600, RRID: AB_10078701) was developed using a synthetic peptide corresponding to amino acid 561–579 of the rat FAAH. It recognizes a dense band at 63 kDa in FAAH recombinant protein (manufacturer data sheet). The antibody has been shown to have specificity in the vervet monkey^46^.

### GFAP

A monoclonal mouse anti-glial fibrillary acidic protein (GFAP clone GA5, Cell Signaling Technology, Danvers, MA, USA, Cat# 3670, RRID: AB_561049) was purified using pig spinal cord GFAP. It is a specific marker of astrocytes, in humans, mice, and rats (manufacturer data sheet). Its specificity has also been verified by immunofluorescence in the marmoset monkey brain^87^.

### NAPE-PLD

A polyclonal rabbit anti-N-acyl phosphatidylethanolamine-specific phospholipase D (NAPE-PLD, Cayman Chemical, Ann Arbor, MI, USA, Cat# 10305, RRID: AB_10507996) was developed using part of a synthetic peptide from human NAPE-PLD. The amino acids (159–172), have been shown to be cross reactive in many species and recognizes an intense band at 46 kDa in human cerebellum tissue, as well as in mouse brain tissue (manufacturer data sheet).

### PV

A monoclonal mouse anti-parvalbumin antibody (PV, Swant, Marly, Fribourg, Switzerland, Cat# 235, RRID: AB_10000343) was developed by hybridization of mouse myeloma cells with spleen cells from mice immunized with parvalbumin purified from carp muscles. PV labels fast-spiking GABAergic interneurons (FSIs)^42,43,88,89^.

### TH

A monoclonal mouse anti-tyrosine hydroxylase antibody (TH clone lnc1, EMD Millipore, Cat# MAB318, RRID: AB_2201528) was developed from tyrosine hydroxylase purified from PC12 cells and recognizes an epitope on the outside of the regulatory N-terminus. It detects TH in many mammalian species, including monkey and human (manufacturer data sheet). Its use has been verified in primates^90^. It was used to stain dopamine-producing cells, located in the shell whose axons originate in the VTA, but not from the SN^20,91^, and in the core to axon projections from both the VTA and SN^20^.

### Data availability

The data generated and analyzed during the current study are available from the corresponding author on request.
